# Sociodemographic Characteristics Associated With an eHealth System Designed to Reduce Depressive Symptoms Among Patients With Breast or Prostate Cancer: Prospective Study

**DOI:** 10.2196/33734

**Published:** 2022-06-08

**Authors:** Nuhamin Gebrewold Petros, Gergo Hadlaczky, Sara Carletto, Sergio Gonzalez Martinez, Luca Ostacoli, Manuel Ottaviano, Björn Meyer, Enzo Pasquale Scilingo, Vladimir Carli

**Affiliations:** 1 National Centre for Suicide Research and Prevention of Mental Ill-Health Department of Learning, Informatics, Ethics and Management Karolinska Institute Stockholm Sweden; 2 Department of Neuroscience "Rita Levi Montalcini" Università degli Studi di Torino Turin Italy; 3 Life Supporting Technologies Universidad Politecnica de Madrid Madrid Spain; 4 Department of Clinical and Biological Sciences Universita degli studi di Torino Turin Italy; 5 GAIA AG Hamburg Germany; 6 Research Center School of Engineering University of Pisa Pisa Italy

**Keywords:** mental health, depression, eHealth, usability, breast cancer, prostate cancer, System Usability Scale, SUS, the user version of the Mobile App Rating Scale, uMARS, Neurobehavioural Predictive and Personalised Modelling of Depressive Symptoms During Primary Somatic Diseases, NEVERMIND system

## Abstract

**Background:**

eHealth interventions have become a topic of interest in the field of mental health owing to their increased coordination and integration of different elements of care, in treating and preventing mental ill health in patients with somatic illnesses. However, poor usability, learnability, and user engagement might affect the effectiveness of an eHealth intervention. Identifying different sociodemographic characteristics that might be associated with higher perceived usability can help improve the usability of eHealth interventions.

**Objective:**

This study aimed to identify the sociodemographic characteristics that might be associated with the perceived usability of the NEVERMIND (Neurobehavioural Predictive and Personalised Modelling of Depressive Symptoms During Primary Somatic Diseases) eHealth system, comprising a mobile app and a sensorized shirt, in reducing comorbid depressive symptoms in patients with breast or prostate cancer.

**Methods:**

The study included a total of 129 patients diagnosed with breast (n=80, 62%) or prostate (n=49, 38%) cancer, who received a fully automated mobile app and sensorized shirt (NEVERMIND system). Sociodemographic data on age, sex, marital status, education level, and employment status were collected at baseline. Usability outcomes included the System Usability Scale (SUS), a subjective measure that covers different aspects of system usability; the user version of the Mobile App Rating Scale (uMARS), a user experience questionnaire; and a usage index, an indicator calculated from the number of days patients used the NEVERMIND system during the study period.

**Results:**

The analysis was based on 108 patients (n=68, 63%, patients with breast cancer and n=40, 37%, patients with prostate cancer) who used the NEVERMIND system for an average of 12 weeks and completed the study. The overall mean SUS score at 12 weeks was 73.4 (SD 12.5), which indicates that the NEVERMIND system has good usability, with no statistical differences among different sociodemographic characteristics. The global uMARS score was 3.8 (SD 0.3), and women rated the app higher than men (β=.16; *P*=.03, 95% CI 0.02-0.3), after adjusting for other covariates. No other sociodemographic characteristics were associated with higher uMARS scores. There was a statistical difference in the use of the NEVERMIND system between women and men. Women had significantly lower use (β=–0.13; *P*=.04, 95% CI −0.25 to −0.01), after adjusting for other covariates.

**Conclusions:**

The findings suggest that the NEVERMIND system has good usability according to the SUS and uMARS scores. There was a higher favorability of mobile apps among women than among men. However, men had significantly higher use of the NEVERMIND system. Despite the small sample size and low variability, there is an indication that the NEVERMIND system does not suffer from the *digital divide*, where certain sociodemographic characteristics are more associated with higher usability.

**Trial Registration:**

German Clinical Trials Register RKS00013391; https://www.drks.de/drks_web/navigate.do?navigationId=trial.HTML&TRIAL_ID=DRKS00013391

## Introduction

### Background

Physical illnesses such as cancer take a toll on a patient’s physical well-being and mental health [1]. The 1-year prevalence of depression is approximately 3% in the general population and between 8% and 24% in patients diagnosed with cancer [2]. Mental ill health, especially depressive symptoms, can affect the quality of life and response to treatment and prognosis in patients diagnosed with breast or prostate cancer, subsequently affecting the prognosis of the cancer outcome [3,4]. Consequently, eHealth and information and communication technology–based self-management tools are of interest to the field of mental health because of their increased engagement with patients, faster response times, and increased coordination and integration of different elements of care [5-7]. These eHealth interventions are designed to curb mental ill health–related consequences at the individual, societal, and health care levels [8-10]. Understanding different aspects of the usability of eHealth interventions could provide substantial clinical benefits [11]. For example, if the technology has poor usability and learnability and low user engagement, the overall effectiveness on patient outcomes may be low, even if the clinical content of the intervention is otherwise effective [12]. Identifying barriers to and facilitators of the implementation process has the potential to streamline eHealth interventions to deliver the intended clinical content optimally. The International Organization for Standardization, an organization that measures and certifies the quality, safety, and efficiency of products, services, and systems, defines usability as “the extent to which a system, product or service can be used by specified users to achieve specified goals with effectiveness, efficiency and satisfaction in a specified context of use” [13]. To determine the usability of any new eHealth technology, rigorously developed and appropriate measures must be chosen [12]. One method of determining usability is to identify the different sociodemographic variables associated with better or higher use of the eHealth intervention, measured using appropriate questionnaires and use data. A recent literature review showed that older age, lower income, lower education, living alone, and living in rural areas were associated with lower eHealth intervention use in patients diagnosed with chronic disorders [14]. It is advantageous to investigate the different sociodemographic characteristics that can specifically influence the use of newly developed eHealth interventions.

### Objectives

The objective of this study was to determine the different sociodemographic characteristics that are associated with the perceived usability of the NEVERMIND (Neurobehavioural Predictive and Personalised Modelling of Depressive Symptoms During Primary Somatic Diseases) system, a newly developed eHealth system for helping patients diagnosed with kidney failure, myocardial infarction, leg amputation, and breast and prostate cancer to self-manage their mental health symptoms, including depressive symptoms. As the NEVERMIND system is a newly developed system, the usability and effectiveness of the system need to be investigated. The aim of our study was to identify different sociodemographic variables that might be associated with the perceived usability of the NEVERMIND system in patients with breast or prostate cancer.

## Methods

### Overview

**Figure 1:**
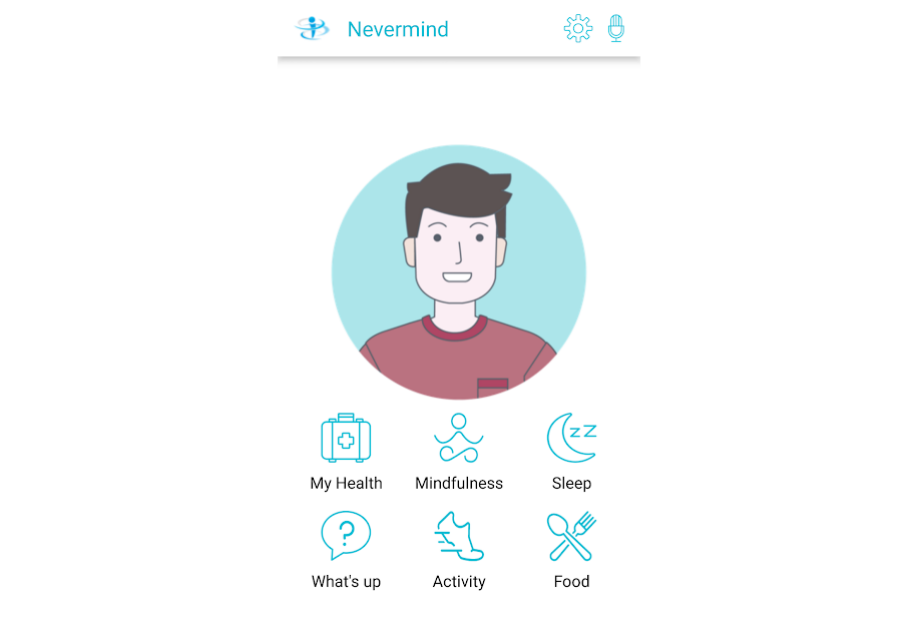
Welcome page of the NEVERMIND (Neurobehavioural Predictive and Personalised Modelling of Depressive Symptoms During Primary Somatic Diseases) mobile app.

**Figure 2:**
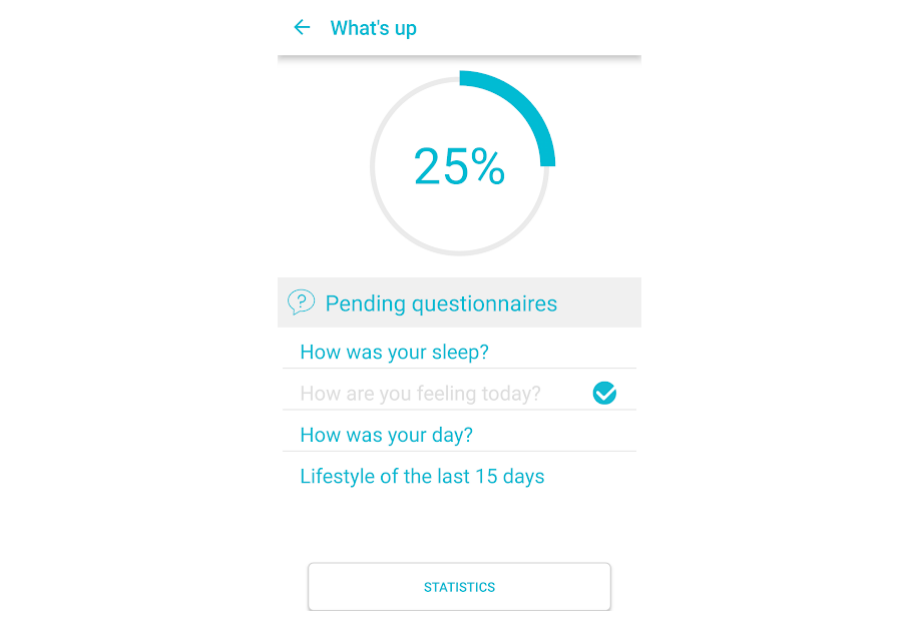
An example of questions administered on the NEVERMIND (Neurobehavioural Predictive and Personalised Modelling of Depressive Symptoms During Primary Somatic Diseases) mobile app.

This study used data from the European Union–funded Horizon 2020 project, NEVERMIND. NEVERMIND uses information and communication technology–enabled self-management procedures. The NEVERMIND system comprises a sensorized shirt to collect biomedical data (electrocardiogram, respiration dynamics, and body movement), and a user interface in the form of a mobile app to collect data on mental health symptoms (depressive and anxiety-related symptoms, stress, and sleep problems) using mood-assessing psychometric questionnaires (Figure 1). Data from the questionnaires and biomedical data are used to predict patients’ depressive symptoms, to provide effective feedback and recommendations (Figure 2). This feedback includes personalized lifestyle behavioral feedback on physical activity, sleep hygiene, dietary habits, mindfulness practice, and cognitive behavior therapy training.

The effectiveness of the NEVERMIND system was evaluated in a randomized controlled trial of 425 patients aged ≥18 years. Patients diagnosed with breast cancer, prostate cancer, myocardial infarction, kidney failure, or leg amputation were recruited from clinical centers in Turin and Pisa, Italy, and Lisbon, Portugal, from November 2017 to December 2019. The results of the randomized controlled trial showed that the NEVERMIND system was superior to standard care in reducing depressive symptoms in patients diagnosed with severe somatic illnesses [15]. In the randomized controlled trial, patients were allocated to either receive the NEVERMIND system in addition to standard care or receive standard care only. Patients in the NEVERMIND intervention received a mobile phone with the NEVERMIND app on it and the sensorized shirt at the recruitment center. The mobile phone that the patients received was configured to use only the app. Patients also completed baseline sociodemographic and usability questionnaires. The usability, acceptability, and satisfaction questionnaires were administered at 2 time points in the study to evaluate specific aspects and assess the subjective quality of the NEVERMIND system. Patients in the NEVERMIND intervention group used the NEVERMIND system for 12 weeks; usability questionnaires were administered at 4 weeks and at the end of 12 weeks.

### Recruitment

The inclusion and exclusion criteria for the NEVERMIND trial are included in the published protocol [10]. The following inclusion and exclusion criteria refer to the selection of patients for this study. In this study, patients with breast or prostate cancer were recruited from the Piedmont Oncological Network at San Luigi Gonzaga University Hospital, Turin, Italy, and the Breast Unit-Oncology Department and Urology Department at Città della Salute e della Scienza University Hospital, Turin, Italy.

### Inclusion Criteria

Patients who were allocated to the NEVERMIND intervention group, had a diagnosis of either breast or prostate cancer, and completed the trial were included in the study.

### Exclusion Criteria

Patients were excluded if they were allocated to the control group in the NEVERMIND study or if they were in the NEVERMIND intervention group but were diagnosed with other severe somatic conditions, such as kidney failure, leg amputation, and myocardial infarction, as the scope of the study was limited to patients diagnosed with cancer. Patients who belonged to the NEVERMIND intervention group but dropped out of the study before receiving the NEVERMIND system were also excluded.

### Data Collection

#### Exposure Variables

Sociodemographic information, including age, sex, marital status, education level, employment status, and living arrangements, was recorded at baseline.

#### Outcome

##### Overview

Three usability metrics were used as the outcome measures to evaluate the usability of the NEVERMIND system:

1. System Usability Scale (SUS): patients completed the SUS at 4 weeks (interim) and 12 weeks (final) after using the NEVERMIND system. The SUS is one of the most frequently used usability measurements that covers the attributes *learnability* and *satisfaction* of the usability dimensions [12,16,17]. The scale is a 10-item subjective measure that can quantify how well users have interacted with and used the product, covering the ease of use of different functionalities, and assessing any technical issues during use, the user’s impression and benefits of using the system. Each item’s score ranges from 0 to 4, and the sum of the items is multiplied by 2.5 to give a transformed composite scale that ranges from 0 to 100; a score of 68 is considered above average [17]. The scale has an interitem correlation of 0.34 to 0.69 and high reliability (Cronbach α=.91) [12]. The SUS is used to assess the usability of eHealth tools in different fields [18,19].
2. The user version of the Mobile App Rating Scale (uMARS): patients completed uMARS at 12 weeks after using the NEVERMIND system. uMARS is the adapted end-user version of the Mobile App Rating Scale, a scale for digital health experts that measures how good a mobile health app is in different dimensions. uMARS measures the app quality by measuring engagement, functionality, aesthetics, and information, to design and develop high-quality mobile apps [20]. The uMARS global score, as well as the 4 objective quality scales, ranges from 0 to 5, with 5 indicating the app to be of very high quality [20]. The uMARS has also been shown to have high internal consistency (Cronbach α=.90) and a good interrater reliability correlation coefficient (0.66-0.70) [20].
3. Use of the NEVERMIND system: the NEVERMIND system uses biomedical data and mental health symptoms gathered from the sensorized shirt and mobile app, respectively, to deliver personalized and timely lifestyle and behavioral feedback as well as mindfulness and cognitive behavioral therapy training in the form of different modules within the mobile app.

The 4 modules are described in the following sections.

##### Physical Activity

**Figure 3:**
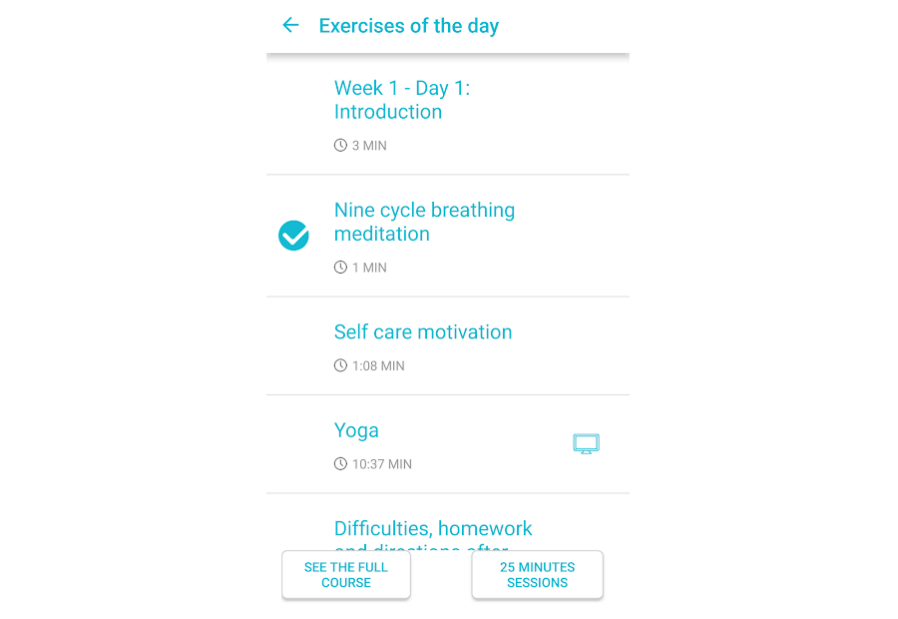
Physical exercise module of the NEVERMIND (Neurobehavioural Predictive and Personalised Modelling of Depressive Symptoms During Primary Somatic Diseases) mobile app.

The physical activity module was designed to reinforce motivation and help the patient achieve goals established at enrollment, based on the patient’s physical functionality and capacity as evaluated by a clinician. Patients had access to a list of suggested exercises in a video format, a list of previously performed exercises, and tips and recommendations guiding them on how to perform the suggested exercises (Figure 3).

##### Dietary Recommendations

Similar to the physical activity module, the dietary module was designed to reinforce motivation and help patients achieve incremental goals. A clinician set these goals by considering the type of diet the patient was following, as well as the dietary preferences of the patient. Patients were recommended types of breakfast they should have and how much protein, carbohydrate, and fat they should consume, among other things. These dietary recommendations were presented in recipe videos and educational reading content.

##### Sleep Hygiene Practice

Patients were instructed to use a sleep agenda and report on parameters related to sleep quality. Upon opening the sleep module, patients were asked about their sleep quality during the previous night (eg, hours in bed and time to fall asleep). Patients were then directed to 4 options: going to bed, daily recommendations on sleep practice, results, and tips. Sleep practice was delivered in video or audio format. Patients were also prompted to wear the sensorized shirt while performing the sleep practice.

##### Mindfulness Practice

The mindfulness module included different types of mindfulness practice of different lengths. The practices offered to patients were personalized according to their disease, preferences set during their enrollment, and mental health symptoms reported in their daily questionnaires. The module also had the option of wearing the sensorized shirt during any of the mindfulness practice sessions. When the user was wearing the shirt, the app received biofeedback consisting of the user’s respiratory and heart rates. This information was then displayed on the screen when the user performed the exercise. The daily recommended practice showed users one practice a day that they should try to complete.

Overall, all the modules were designed to reinforce motivation, help achieve the intended goal and in turn, improve the patient’s mental health, including depressive symptoms. Each module had use data recorded by distinct days of use (the number of days a patient has used the specific module), log data (when a patient opens the app but does not necessarily use the app or the modules or sends any data to the server), and the number of completed practices (the number of completed practices within each module). A remote server also collected data from the sensorized shirt, where use data were expressed in terms of distinct days of use and log data.

Our usage index was computed by adding the number of days patients used only the app or only the shirt and days that patients used both the app and the shirt. However, as patients had different study periods, we divided the index by the number of days patients were included in the study.

### Statistical Analysis

The sample size was based on the number of patients with breast or prostate cancer in the NEVERMIND study who received the NEVERMIND system. All outcomes were measured on a continuous scale, and sociodemographic characteristics were dichotomized. The variable, living arrangement, was categorized as either living alone or being a cohabitant (living with a partner, other family members, or with other people), whereas education was dichotomized as low (no education and primary or secondary school) and high (college level and above). Marital status was also dichotomized as single (unattached, divorced, separated, and widowed) and living with someone (marriage or domestic partnership), whereas employment status was categorized as either unemployed (retired, unemployed, or not working owing to other reasons, including ill health) or employed. The normality of the outcomes was checked using the Skewness and Kurtosis tests. The association between sociodemographic characteristics and SUS, uMARS, and usage index was measured using multivariate regression. All analyses were performed using STATA/MP (version 15.1; StataCorp LLC).

### Ethics Approval

Ethical approval for the NEVERMIND study was submitted and approved by each of the local research ethics committees in the centers where the intervention was implemented (Pisa Comitato Etico di Area Vasta Nord Ovest (Comitato Etico Sperimentazione Farmaco; 912/2015); Ethical Committee of Città della Salute e della Scienza di Torino University Hospital and Ethical Committee of San Luigi Gonzaga University Hospital, Orbassano (185/2015); Ethics Committee of the Medical Academic Centre of the University of Lisbon (223/16). Additional ethical approval for the analysis of the pseudoanonymized data was obtained by the Swedish Ethics Review Authority (Etikprövningsmyndigheten; Dnr 2020-04175).

## Results

### Overview

A total of 129 patients diagnosed with either breast or prostate cancer were included in the intervention group. Of the 129 patients, 108 (83.7%) completed the study and 21 (16.3%) dropped out after the baseline assessment. Of these 21 patients, 11 (52%) dropped out before receiving the NEVERMIND system because of nickel allergy (1/21, 5%), pacemaker (1/21, 5%), emergency surgery (2/21, 10%) and not coming back to get the system (7/21, 33%). Of the remaining 21 patients, 9 (43%) received the system but did not open the mobile app or use the shirt and 1 (1/21, 5%) completed the intervention without outcome data. There were no statistically significant baseline differences between patients who completed the study and those who dropped out. Of the 108 patients who completed the study, 40 (37%) patients were men, and 68 (63%) patients were women, with a mean age of 58.6 (SD 9.3; range 34-74) years (Table 1). Most patients lived with someone (93/108, 86.1%), were highly educated (87/108, 80.6%), and were in a partnership (78/108, 72.2%). Patients were instructed to use the NEVERMIND system for a total of 12 weeks. However, the average number of days of use in the NEVERMIND study was 44.9 days, which is approximately 6 weeks, and only 12 patients had used the NEVERMIND system for the recommended period of ≥12 weeks (data not shown).

**Table 1:** Multivariate regression of sociodemographic characteristics and the System Usability Scale (SUS) score at 4 weeks (interim) and 12 weeks (final).

		Participants, n (%)	SUS (interim)			SUS (final)	
			Value, mean (SD)^a^	β coefficient (95% CI)	Value, mean (SD)^b^		β coefficient (95% CI)
Total		108 (100)	70.9 (12.3)	N/A^c^	73.4 (12.5)		N/A
**Sex**							
	Men (reference)	40 (37)	72.01 (11)	−2.88 (−8.6 to 2.8)	72.1 (13.6)		−0.31 (−5.8 to 5.2)
	Women	68 (63)	70.4 (13)	N/A	74.2 (11.9)		N/A
Age (years), mean (SD)		58.6 (9.3)	N/A	−0.08 (−0.4 to 0.2)	N/A		−0.24 (−0.5 to 0.1)
**Living arrangement**							
	Living alone (reference)	15 (13.8)	71.1 (14.2)	2.77 (−6.9 to 12.5)	69.5 (11.8)		6.57 (−3.1 to 16.2)
	Cohabitant	93 (86.1)	70.9 (12.1)	N/A	74.0 (12.6)		N/A
**Education**							
	Low (reference)	21 (19.4)	67.8 (11.8)	4.64 (−1.7 to 11.0)	70.4 (11.2)		4.26 (−1.96 to 10.5)
	High	87 (80.5)	71.7 (12.4)	N/A	74.2 (12.8)		N/A
**Marital status**							
	Single (reference)	30 (25.9)	72.1 (11.8)	−3.66 (−10 to 3.7)	73.2 (11.1)		−3.09 (−10.4 to 4.2)
	Married	78 (72.2)	70.5 (12.5)	N/A	73.5 (13.1)		N/A
**Employment**							
	Unemployed (reference)	56 (51.9)	70.2 (11.9)	.30 (−5.3 to 5.9)	72.2 (13.3)		−0.50 (−6.0 to 4.9)
	Employed	52 (44.4)	71.7 (12.7)	N/A	74.7 (11.7)		N/A

^a^n=104.

^b^n=107.

^c^N/A: not applicable.

### SUS Score

Table 1 shows how different sociodemographic characteristics are related to the SUS score described in a multivariate regression model using the β coefficient and 95% CI. All patients except one (107/108, 99.1%) had data for the SUS at the final follow-up, and 3.7% (4/108) of the patients had missing SUS scores at the interim time point. The mean SUS score at the final time point (mean 73.4, SD 12.5) was higher than the mean SUS score at the interim time point (mean 70.9, SD 12.3; Table 1). However, there were 3 more patients when computing the SUS at the final time point than at the interim time point; the mean SUS score at the final time point, excluding the scores of the patients those who had missing SUS scores at the interim time point, was 73.3 (data not shown).

The mean SUS score at the final time point was normally distributed based on the Skewness and Kurtosis tests (*P*=.05), whereas the mean SUS score at the interim time point was not normally distributed (*P*=.001); thus, a nonparametric regression was appropriate. However, both parametric and nonparametric regressions yielded similar β estimates, with slightly different CIs. Age was significantly associated with SUS score at the final time point in a univariate model (*P*=.04, data not shown), but it became insignificant in a multivariate model after adjusting for other covariates (*P*=.15, data not shown). No other sociodemographic characteristics were associated with a higher SUS score at either the interim or final time points.

### uMARS Score

At 12 weeks, 107 patients completed the uMARS. The global uMARS score was 3.8 (Table 2), which is above the average (3.0) for this scale. Each subscale scored above average, with engagement, functionality, aesthetics, and information scoring at 3.5, 3.9, 3.6, and 4.2, respectively (data not shown). Table 2 shows how different sociodemographic characteristics were related to uMARS scores. A Skewness and Kurtosis test showed that the uMARS was normally distributed (*P*=.54, data not shown).

The mean uMARS score was significantly higher for women (mean 3.9, SD 0.3) than that for men (mean 3.7, SD 0.3). No sociodemographic characteristics were associated, either in the univariate analyses or in the multivariate model, with different uMARS scales, except for women rating the app higher than men (*P*=.03; Table 2). A further investigation into the uMARS showed that the subscale *engagement* showed significant differences between women and men (β=0.26; *P*=.02, 95% CI 0.04-0.48). Women had a mean engagement score of 3.64 (SD 0.52; range 2.4-5), whereas men had a mean engagement score of 3.36 (SD 9.51; range 2.2-4.4; data not shown). There were no significant differences in the other subscales.

**Table 2:** Multivariate regression of sociodemographic characteristics and the user version of the Mobile App Rating Scale score at 12 weeks (final).

Sociodemographic characteristics	β coefficient (SE; 95% CI)	*P* value
Sex	0.16 (0.07; 0.02 to 0.30)	.03^a^
Age (years)	−0.003 (0.004; −0.01 to 0.005)	.43
Marital status	−0.02 (0.09; −0.02 to 0.17)	.83
Living arrangement	0.02 (0.13; −0.23 to 0.27)	.86
Education level	0.02 (0.08; −0.14 to 0.17)	.82
Employment status	−0.07 (0.07; −0.21 to 0.07)	.30

^a^Statistically significant at *P*<.05.

### Use of the NEVERMIND System

A total of 99.1% (107/108) of patients had log data for computing the usage index of the NEVERMIND system. The mean usage index was 0.48. The usage index was not normally distributed according to the Skewness and Kurtosis tests (*P*<.001); however, most patients (73/107, 68.2%) had a usage index higher than the mean. However, the distribution was because of a few outliers, and both parametric and nonparametric regressions yielded similar effect estimates. Table 3 shows the results of parametric regression.

No sociodemographic characteristics had statistically significant associations with higher or lower use of the system, except for women, showing a lower usage index than men. The mean usage index for women was 0.43 (SD 0.28; range 0.02-0.99), whereas that for men was 0.56 (SD 0.24; range 0.04-0.97; data not shown). Women had significantly lower use of the NEVERMIND system during the study period.

**Table 3:** Multivariate regression of sociodemographic characteristics and use of the NEVERMIND (Neurobehavioural Predictive and Personalised Modelling of Depressive Symptoms During Primary Somatic Diseases) system at 12 weeks (final).

Sociodemographic characteristics	β coefficient (SE; 95% CI)	*P* value
Sex	−0.13 (0.06; −0.25 to −0.01)	.04^a^
Age (years)	−0.001 (0.003; −0.01 to 0.01)	.82
Marital status	0.14 (0.08; −0.02 to 0.30)	.09
Living arrangement	−0.11 (0.10; −0.32 to 0.09)	.28
Education level	0.05 (0.07; −0.09 to 0.20)	.49
Employment status	−0.05 (0.06; −0.17 to 0.07)	.42

^a^Statistically significant at *P*<.05.

## Discussion

### Principal Findings

This study aimed to investigate the different sociodemographic characteristics that can determine higher usability, as measured by 3 usability outcomes. We found that none of the sociodemographic characteristics investigated were associated with different types of usability outcomes except for women rating the mobile app higher on the uMARS and that men having used the system more than women. Several methods exist to measure the usability of systems [12,21]. In our study, we used subjective measurements in tandem with use metrics; this is considered to be a more reliable predictor of use frequency than using subjective scales or logging tasks alone [21].

The patients had a higher SUS score at the final than at the interim time point. The higher SUS score at the final time point could be an experience effect, that is, with more time and opportunity to navigate through the app, patients could have gained app-relevant experience and skills, thereby increasing the app usability. This especially aligns with the SUS, as it comprised 10 statements covering the need for support and training, and complexity of the system—aspects that improve over time.

The SUS enables us to compare our system with other comparable and highly thought of products that serve similar purposes and possibly cater to the same group of users, such as patients affected by other somatic illnesses. For example, Grossert et al [22] reported the usability of a web-based Stress Management Intervention (STREAM) in 11 patients diagnosed with cancer, of whom 4 (36%) were diagnosed with breast cancer and 1 (9%) with prostate cancer. They found the overall SUS score of STREAM to be 83.6, which was higher than the predefined cutoff for good usability and the NEVERMIND system. However, the NEVERMIND system was tested with more patients and was geared mainly toward reducing depressive symptoms. There is a paucity of research on the evaluation of eHealth systems using SUS in patients with cancer in the field of mental health.

uMARS was only administered at the final evaluation, and the global score was recorded as 3.8, with the subscale information scoring very high at 4.2. Being a woman was the only sociodemographic characteristic associated with a higher uMARS score. Research shows that women have higher health care–seeking behavior, especially when it comes to mental health care [23], which can lead to higher engagement with the mobile app.

The uMARS is also widely used to evaluate different mobile apps geared toward patients diagnosed with cancer. A recent systematic descriptive search conducted by Amor-García et al [24] analyzed 46 apps available for patients diagnosed with different types of cancer, including prostate cancer. They found that the mean Mobile Appl Rating Scale score of these 46 apps was 2.98, with 13 apps scoring ≥3.5. In another evaluation of mobile apps designed for patients diagnosed with cancer, including patients with breast or prostate cancer, Böhme et al [25] reported a significantly lower score (1.96). Interestingly, these previous studies also noted the engagement of patients to be the lowest scored subscale, similar to what we observed in our sample group. As one of the goals of self-management eHealth tools is to increase the engagement of patients in managing their health, more work is needed in this area.

The usage index metric was used as a quantitative usability measure by looking at how many patients used the sensorized shirt and mobile app until the end of the study. A usage index of 1 indicates that the patient has used either the mobile app or the sensorized shirt or both at least once a day for the duration of the study. Similar to the uMARS scale, this study showed that there was a difference of usage between women and men. Our results showed that women used the system less frequently during the study period. Although the system comprised a sensorized shirt and mobile app, patients were instructed to use the shirt twice a week, with the mobile app being intended for everyday use. Research has indicated that women interact with and use specific types of mobile health apps that are geared toward nutrition and self-care, whereas men interact more with physical activity–related mobile health apps [26]. Therefore, the higher use among men might be related to the contents of the NEVERMIND system, which might have a more engaging physical activity module than the dietary recommendation and mindfulness modules.

### Limitations

This study had some limitations. First, the sample size used for the study was based on the initial sample size calculated to test the clinical effectiveness of the NEVERMIND system, which made it impossible to include more patients. Consequently, the generalizability of the results of this study is limited with respect to giving a definitive conclusion regarding the association between sociodemographic characteristics and usability. However, our results can provide an indication of how sociodemographic characteristics might be associated with usability, which has been documented in previous research. Furthermore, other variables, such as digital literacy and the ability to use these types of technologies, were not included, which can also influence the generalizability of the results to other populations who might have different starting digital literacy despite having similar sociodemographic characteristics. Our study found that sex was associated with differential uMARS scores and use of the system, which inadvertently fully aligns with the cancer diagnosis; that is, all patients diagnosed with breast cancer were women and all patients diagnosed with prostate cancer were men. However, there was no other information available to differentiate sex from cancer diagnosis. The length of use of the NEVERMIND system is another limitation. Ideally, all patients should have used the system for 12 weeks, which was the recommended time in the NEVERMIND study and might have influenced how patients rated the system. Owing to the small sample size, it was not possible to analyze only those who used the system for 12 weeks or more. Another limitation is the choice of outcome measures. For example, the usage index metric does not provide information about the number of tasks completed, time on a task, or error on a task, all of which are important predictors for usability evaluation in eHealth [27]. Thus, the use metric is only a partial indicator of usability. However, using subjective usability measures, such as the SUS and uMARS, coupled with use metrics, contributes to a better benchmark for usability evaluation.

### Conclusions

Research demonstrates that different sociodemographic characteristics are associated with higher use and efficacy of eHealth interventions. Despite the limitations of the study, our initial findings suggest that the usability of the NEVERMIND system does not suffer from a large *digital divide* where certain sociodemographic characteristics are more associated with higher usability. There seems to be an indication that there is higher favorability of the mobile app among women but that men use the NEVERMIND system more. Future research will focus on examining specific modules separately in the NEVERMIND system to understand content-related differences in usability.

## Abbreviations

NEVERMIND: Neurobehavioural Predictive and Personalised Modelling of Depressive Symptoms During Primary Somatic Diseases
SUS: System Usability Scale
uMARS: user version of the Mobile App Rating Scale
